# The cellular response to drug perturbation is limited: comparison of large-scale chemogenomic fitness signatures

**DOI:** 10.1186/s12864-022-08395-x

**Published:** 2022-03-11

**Authors:** Marjan Barazandeh, Divya Kriti, Corey Nislow, Guri Giaever

**Affiliations:** 1 Faculty of Pharmaceutical Sciences, University of British Columbia, 2405 Wesbrook Mall, Vancouver, BC V6T 1Z3 Canada; 2 Department of Biochemistry & Molecular Biology, Faculty of Medicine, Life Sciences Centre, 2350 Health Sciences Mall, Vancouver, BC V6T 1Z3 Canada

**Keywords:** Genomics, Chemogenomics, Fitness assay, Reproducibility, *Saccharomyces cerevisiae*, Drug target, Mechanism of action, HIPHOP

## Abstract

**Background:**

Chemogenomic profiling is a powerful approach for understanding the genome-wide cellular response to small molecules. First developed in *Saccharomyces cerevisiae*, chemogenomic screens provide direct, unbiased identification of drug target candidates as well as genes required for drug resistance. While many laboratories have performed chemogenomic fitness assays, few have been assessed for reproducibility and accuracy. Here we analyze the two largest independent yeast chemogenomic datasets comprising over 35 million gene-drug interactions and more than 6000 unique chemogenomic profiles; the first from our own academic laboratory (HIPLAB) and the second from the Novartis Institute of Biomedical Research (NIBR).

**Results:**

Despite substantial differences in experimental and analytical pipelines, the combined datasets revealed robust chemogenomic response signatures, characterized by gene signatures, enrichment for biological processes and mechanisms of drug action. We previously reported that the cellular response to small molecules is limited and can be described by a network of 45 chemogenomic signatures. In the present study, we show that the majority of these signatures (66%) are also found in the companion dataset, providing further support for their biological relevance as conserved systems-level, small molecule response systems.

**Conclusions:**

Our results demonstrate the robustness of chemogenomic fitness profiling in yeast, while offering guidelines for performing other high-dimensional comparisons including parallel CRISPR screens in mammalian cells.

**Supplementary Information:**

The online version contains supplementary material available at 10.1186/s12864-022-08395-x.

## Background

A persistent challenge in drug discovery is the validation of the molecular targets and the target pathways that are modulated by bioactive small molecules. This is especially true for target-based approaches where drug candidates selected from high throughput biochemical screens produce unexpected effects when tested in cells and in vivo (e.g. [1]). The consequences can be substantial, for instance when drugs fail in the clinic because of incomplete characterization of their effects in vivo (e.g. [2]). Perhaps, as a consequence, phenotypic, cell-based screens have seen renewed interest (for review see [3]). Yet, despite advances in the complexity and sophistication of phenotypic screens (e.g. [4, 5]), the unambiguous assessment of a drug’s primary, secondary and tertiary effects, in vivo, remains a significant challenge. Successful implementation of chemogenomic assays and an analytical framework would help bridge the gap between bioactive compound discovery and drug target validation.

Chemogenomics integrates drug discovery and target identification through the detection and analysis of chemical-genetic interactions. Despite the increase in such studies, most chemogenomic methods currently rely on correlation to infer drug-target interactions; i.e. few directly identify drug-target chemical-genetic interactions [6]. For example, genome-wide differential expression analysis (aka transcriptomics) is one strategy used to probe Mechanism of Action (MoA) [7–9]. In these studies, gene expression changes induced by chemical perturbation are compared to a compendium of profiles with known drug-target pairs. A drug target can then be inferred based on the similarity of a query profile to an expression profile in the reference compendium based on the “guilt- by-association” principle [10]. Such approaches, while having been greatly expanded in the past decade, still depend on the composition and quality of their reference database and are therefore prone to systematic bias and lab-to-lab variations. Further complicating differential expression approaches is the fact that a genetic knockdown or knockout often lacks a discrete phenotype but nevertheless results in the differential expression of hundreds or thousands of transcripts. In contrast, drug perturbation of the proteins encoded by a locus (or loci) of interest is consequential. By way of example, nocodazole treatment will depolymerize microtubules composed of multiple tubulin isoforms and thereby result in a phenotype, whereas genetic perturbation of a single isoform may have no effect.

Encouragingly, several large-scale efforts to extend chemogenomic assays to mammalian cells have been launched. Notable international efforts to gather and synthesize similar screening data from mammalian cells initially focused on genome-wide siRNAs screens [11, 12]. More recently, CRISPR-based approaches for genome-wide screens (e.g. [13, 14]) have accelerated the collection of chemogenomic interaction data for mammalian systems. Key consortia developed to progress these efforts include; BioGRID (https://orcs.thebiogrid.org/), PRISM (https://www.theprismlab.org/), LINCS (https://lincsproject.org/LINCS/), and DepMAP (https://depmap.org/portal/). These resources and their extensive publication lists provide complementary data on diverse cell lines, cultured in a variety of environmental conditions and challenged with both focused and broad-based chemical libraries. The work of research teams is essential to constrain the “combinatorial explosion of data” from collecting such multidimensional data, which must be annotated and curated to be useful to the scientific community.

In light of the rapid development of mammalian chemogenomics, we sought to establish the scale, scope and reproducibility of the two largest yeast chemogenomic datasets. These functional genetic screens provide mechanistic insight, reporting all chemical-genetic interactions required for drug resistance. Specifically, the HaploInsufficiency Profiling and HOmozygous Profiling (HIPHOP) platform [15, 16] employs the barcoded heterozygous and homozygous yeast knockout collections. HIP exploits drug-induced haploinsufficiency; a phenotype where strain-specific sensitivity (decreased growth rate) is observed in a heterozygous strain deleted for one copy of an essential gene upon exposure to a drug targeting the product of this gene. In HIP, the 20 bp molecular identifiers, unique to each strain, allow the ~1100 essential heterozygous deletion strains to be grown competitively in a single pool and fitness to be quantified by barcode sequencing. The resulting fitness defect (FD) scores report the relative abundance, and therefore the drug sensitivity of each strain. Those heterozygous strains with the greatest FD scores identify the most likely drug target candidates. Similarly, the complementary HOP assay interrogates ~ 4800 nonessential homozygous deletion strains; and identifies genes involved in the drug target biological pathway and those required for drug resistance. The combined HIPHOP chemogenomic profile, reporting drug-target candidates in the HIP assay and genes required for resistance in the HOP assay, provides a comprehensive genome-wide view of the cellular response to a specific compound [5, 6].

We compared a HIPHOP dataset generated in our lab (aka HIPLAB) [16] to one generated by a group at the Novartis Institute for Biomedical Research (NIBR) [17]. The datasets are distinct; they were obtained from two independent platforms, using different experimental designs and distinct analytic pipelines (Table 1). The aims of this study were to assess their reproducibility and the data concordance at different levels of analysis. By analyzing the NIBR and HIPLAB datasets in parallel, we suggest that they may be more accessible to the research community. An additional aim was to identify any biological themes emergent in the combined data that were not obvious from either of the individual datasets.

**Table 1:** Experimental and analytical pipelines of the HIPLAB and NIBR datasets

	HIPLAB	NIBR
Number of screens	3356	2725
Number of unique compounds	3250	1776
Number of HET strains	1095-essential	5796-essential+nonessential
Number of HOM strains	4810	4520
Bioassay	IC20	IC30
HIPHOP assay plates/media	48-well/700ul YPD	24-well/1600ul YPD
experiments per plate	42 drug-treated samples + 6 negative controls (1% DMSO)	10 drug-treated samples in duplicates + 2 negative controls (no drug) + 1 positive control (Benomyl) + 1 contamination control (no cells)
HIPHOP assay device	Tecan Genios spectrophotometer	Cytomat Robotic shaking incubator
starting number of cells	O.D._600_ of 0.02 (~ 400 and ~ 200 cells/strain for HIP and HOP respectively)	100ul and 110ul of a 1.5 O.D._600_ /ml culture (~ 600 and ~ 700 cells/strain for HIP and HOP, respectively
Frequency of Optical Density (O.D.) measurement	15’	60’
Collection time	log-phase cells; 20 and 5 generations for HIP and HOP, respectively	saturated cells; ~ 20 and ~ 5 generations for HIP and HOP, respectively
Final strain intensity value	‘best tag’: tag with the lowest robust coefficient of variation in the control arrays	average of uptag and downtag intensities
z-score calculation for strain_i_ in screen_j_	log_2_ ratio_ij_ = log_2_(median signal from controls/signal from drug-treated sample)	log_2_ ratio_ij_ = log_2_(average signal from replicates of drug-treated samples/average signal from controls sample)
	z scores = FD_ij_ = MADL = (log_2_ ratio_ij_ - median of log_2_ ratio_j_) / MAD log2 ratio screen_j_	Sensitivity scores = FD_ij_ = MADL = (log_2_ ratio_ij_ - median of log_2_ ratio_j_) / MAD log2 ratio screen_j_
		Adjusted MADL based on the variability between replicates: a_MADL_ = min(0.05/p,1)*MADL
		z scores = a_MADL_/standard deviation of a_MADL_ values of strain i over n screens
Significant chemical-genetic interactions	standard normal distribution *P* ≤ 0.001	z-score < -5
Clustering method	Ward hierarchical clustering with dynamic branch cutting	Average-linkage two-way hierarchical clustering

Our comparison shows excellent agreement between chemogenomic profiles for established compounds and correlations between entirely novel compounds. We characterize global properties common to both datasets, including specific drug targets, correlation between chemical profiles with similar mechanism and cofitness between genes with similar biological function. Unique features of each dataset were also uncovered. In our previous report, we identified 45 major cellular response signatures [16]. We also hypothesized that these 45 signatures were comprehensive because, in our simulations, we found that 80% of these clusters would have been identified after screening < 30% of the ~3200 compounds. In the analysis presented here, we found that the majority of these signatures (66.7%) are also present in the NIBR dataset. In addition, by combining the two datasets we were able to: 1) identify robust chemogenomic responses both common and research site-specific, the majority (81%) enriched for Gene Ontology (GO) biological processes and associated with gene signatures, 2) infer chemical diversity/structure, and 3) gauge screen-to-screen reproducibility within replicates and between compounds with similar MoA. We present the data in an interactive web application that provides a resource for the discovery of functional interactions between genes, compounds and biological processes (Comparative chemogenomics).

## Results and discussion

### Overview of NIBR and HIPLAB screens

Because all the comparisons are based on the ability to compare both datasets, we describe each dataset in detail. The data processing strategies of the raw data were fundamentally different between the two research sites (Table 1). In the HIPLAB dataset, the raw data was normalized separately for the strain-specific uptags and downtags and independently for the heterozygous essential and homozygous nonessential strains, creating 4 sets of results: uptag/het, uptag/hom, downtag/het, downtag/hom. For each set, logged raw average intensities were normalized across all arrays using a variation of median polish that incorporates batch effect correction [16]. Because the performance of the two tags in each strain can vary significantly, a ‘best tag’ was identified for each strain, defined as the tag with the lowest robust coefficient of variation across all of the control microarrays. For each array, tags were removed if they did not pass the computed compound and control background thresholds, calculated from the median + 5MADs of the raw signal from the unnormalized intensity values of the used (corresponding to strain tags) and unused (control) features on the array across all arrays. In the NIBR dataset, arrays were normalized by “study id” (a set of ~40 compounds), but were not corrected for batch effects. NIBR tags that performed poorly based on the correlation values of uptags and downtags across different intensity ranges in the control arrays were removed and the remaining tags were *averaged* to obtain strain intensity values.

In the HIPLAB dataset, relative strain abundance was quantified for each strain as the log_2_ of the median signal in the control condition divided by the signal from the compound treatment. The final FD score was expressed as a robust z-score where the median of the log_2_ ratios for all strains in a given screen is subtracted from the log_2_ ratio of a specific strain and divided by the MAD of all log_2_ ratios for all strains in that screen. In the NIBR dataset, the inverse log_2_ratio_HIPLAB_ was used with three differences: 1) average intensities of controls were used (instead of median signals), and 2) the average of signals of the compound samples across replicates was used instead of a single value (Table 1), and 3) the final gene-wise z-score was normalized for median and standard deviation of each strain across all experiments using quantile estimates (see Methods).

Both laboratories constructed pools of heterozygous and homozygous strains in a similar manner and collected samples robotically for both the HIP and HOP assays as previously described [18]. For NIBR experiments, samples were collected at fixed time points (which served as a proxy for the number of cell doublings), whereas in the HIPLAB experiments cells were collected based on the actual doubling time. Notably, in the NIBR pools, ~300 fewer homozygous deletion strains were detectable compared to the HIPLAB pools. These strains correspond to known slow-growing deletions [19] and their absence is likely due to the fact that the pool was allowed to grow overnight (~16hrs) in the NIBR assays.

Another difference between protocols was that NIBR screened all heterozygous strains, deleted for both essential and nonessential genes, while the HIPLAB screened only the essential heterozygotes. We did not screen nonessential heterozygotes based on the rationale that because the HIP assay requires a drug-induced fitness effect, the reduction in gene-dosage of nonessential genes (which are not required for growth) should not produce a phenotype [20]. Indeed, in practice the HIP profiles of the nonessential heterozygotes do not correlate with nonessential homozygotes for the same drug, and these nonessential heterozygote profiles are not biologically informative. For example, In HOP screens of nonessential deletion strains, RAD genes have high FD scores in the presence of a DNA-damaging agent (mechlorethamine), but none of these strains were sensitive as heterozygotes (Fig. S1). The exception to this is the small number of nonessential heterozygous strains that exhibit severe fitness defects as homozygotes. As these strains exhibit ‘nearly essential’ phenotypes as homozygotes, they would be expected to exhibit drug-induced haploinsufficiency as heterozygotes and therefore could be included in the HIP assay.

We next compared the depth and breadth of each screening dataset. The NIBR screening library included 1641 blinded, propriety compounds and 135 unblinded reference compounds with known mechanisms of action. In total there were 2956 HIP and 2923 HOP screens against 1776 discrete chemical structures. Because we wanted to combine the essential HIP data with the HOP data, we took the 2725 compounds common to both datasets, resulting in 1771 distinct compounds and a decrease in the number of data points from ~30 million to ~15 million. ~45% of the NIBR screening library, (representing 1228 compounds, 491 distinct) could practically be considered replicate screens because they exhibit correlations on par with the true replicates, despite being screened at different concentrations. For example, we observe such “practical replicates” when a particular compound is screened at a different concentration, yet the level of inhibition is comparable. Supporting this observation, the majority of such “replicates” clustered together with the same compound despite being screened at a different concentration (~65%). After excluding replicates, the NIBR screening library comprised 2725 HIPHOP screens against 1771 unique compounds resulting in ~9 million independent data points. The HIPLAB screening library comprised 3356 screens and 3250 unique compounds selected from a set of > 50,000 maximally diverse small molecules (~ 20 million independent data points) with unknown mechanisms and ~200 characterized drugs or chemical probes.

The structural diversity of the screening libraries reflects the large size of the screening efforts. While NIBR did not provide the compound structures of their libraries, they reported that 50% of the pairwise comparisons between compounds had Tanimoto coefficients less than 0.1 [17]. In comparison, the HIPLAB compounds were of lower diversity; ~43% of the pairwise comparisons had Tanimoto coefficients less than 0.1. Because the NIBR structures were not provided (with the exception of 135 reference compounds and 15 novel inhibitors), this claim is not verifiable [17].

### Coinhibition between chemogenomic profiles

**Fig. 1:**
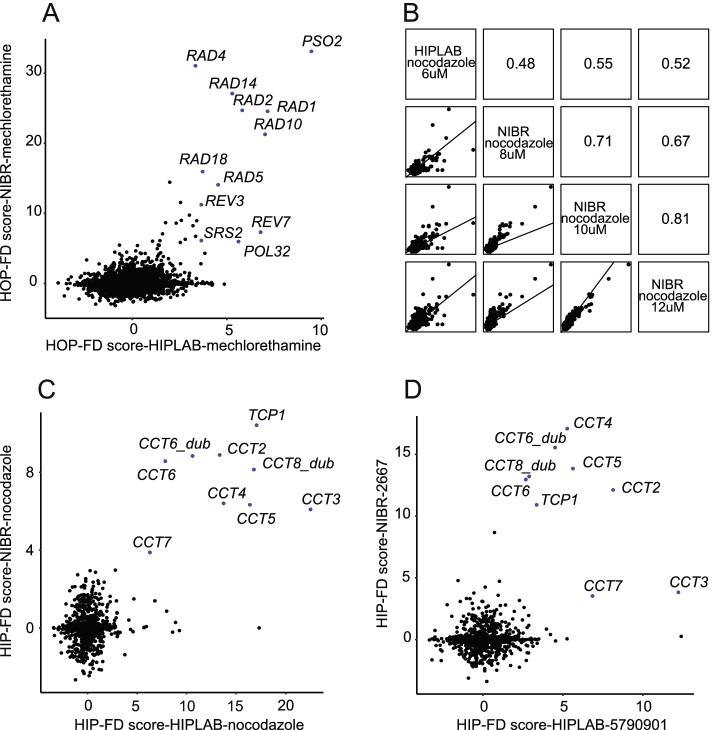
Coinhibition between chemogenomic profiles. **A** Gene-level view of significant FDs observed in the HOP assay for mechlorethamine in NIBR and HIPLAB datasets. **B** Pairwise comparisons of chemical profiles for nocodazole for NIBR and HIPLAB datasets. Pearson coinhibition value is indicated. Gene-level view of significant FDs observed in the HIP assay for (**C**) nocodazole and (**D**) between two novel compounds, UBC 5790901 and NIBR 2667

To compare the HIPLAB and NIBR screens, we selected ~150 chemogenomic profiles representing ~50 reference compounds with known MoA that were screened by both NIBR and HIPLAB (Table 2). For many of these compounds, the drug target is well-established in yeast. Chemogenomic profiles were compared individually using ‘coinhibition’ values, where coinhibition is defined as the degree of similarity between two chemogenomic profiles, i.e., the FD scores across all genes in each screen, using Pearson correlation as a metric. By way of example, the HOP profiles for the mechlorethamine, a DNA-damaging agent, identified a similar set of DNA repair genes including *RAD1*, *RAD2*, *RAD4*, *RAD5*, *RAD10*, *RAD14*, *RAD18*, *REV7*, *REV3*, *SRS2* and *PSO2* (Fig. 1A). A pairwise comparison of four nocodazole chemogenomic profiles exhibiting between-drug correlations of 0.48 (and greater) across the entire set of deletion strains (Fig. 1B) showed the correlation values increased when comparing only those individual genes exhibiting significant FD scores in the NIBR and HIPLAB in the HIP nocodazole profiles (Fig. 1C). In this case, the HIP genes identified are enriched for genes required for tubulin folding (*CCT* genes). Finally, based on a correlation value of >0.5 with the nocodazole profiles, we found that the HIP profiles of two novel compounds, NIBR 2667 and HIPLAB 5790901, identify a nearly identical set of genes (Fig. 1D), suggesting they operate using a similar MoA. It should be noted that because the screens were performed at different concentrations, a linear correlation of one is not expected.

**Table 2:** Mean coinhibition by drug mechanism

Drug class (cluster)	Compounds	# of compounds	Mean coinhibition
Microtubule depolymerizers	benomyl; nocodazole	5	0.73
Dfr1 inhibitors	methotrexate	8	0.62
Ribonucleotide reductase inhibitors	hydroxyurea	3	0.62
Ionophores	nigericin	4	0.61
Metabolites	sphingosine	4	0.61
Fas1 inhibitors	cerulenin; 1-3 diallylurea	3	0.51
Dopamine antagonists	chlorpromazine; trifluoperazine	4	0.48
mTOR inhibitors	rapamycin; caffeine	14	0.45
Calcium channel blockers	amiodarone	3	0.47
Acc1 inhibitors	tofa	3	0.47
Antineoplastic antibiotics	bleomycin	3	0.45
Iron chelators	curcumin	10	0.45
Statins	atorvastatin; cerivastatin; fluvastatin; itavastatin	6	0.44
Aclacinomycin antibiotics	aclarubicin	2	0.42
Cell wall inhibitors	caspofungin; ergokonin	6	0.42
Lcb1/Lcb2 inhibitors	myriocin	2	0.41
FK506; calcineurin inhibitors	ascomycin; tacrolimus	2	0.40
DNA topoisomerase I inhibitors	camptothecin	3	0.38
N-linked glycosylation inhibitors	tunicamycin	4	0.38
Translation inhibitors	anisomycin; cycloheximide	4	0.37
Azoles	clotrimazole; cyproconazole; fluconazole; myclobutanil; voriconazole	6	0.36
Anthracycline antibiotics	daunorubicin; doxorubicin; epirubicin; ravidomycin	11	0.35
Erg1 inhibitors	butenafine; naftitine; terbinafine	6	0.34
Pyrimidine antagonists	5-fluorouracil; flucytosine; 5-fluorouridine; carmofur; 5-fluorocytosine	7	0.31
Erg2 inhibitors	alverine citrate; dyclonine; fenpropimorph; haloperidol	9	0.28
Transcriptional elongation inhibitors	6-azauridine; 6-azauracil; mycophenolic acid	11	0.25
Alkylating agents	mechlorethamine; melphalan; methyl methanesulfonate; mitomycin C	2	0.24

**Fig. 2:**
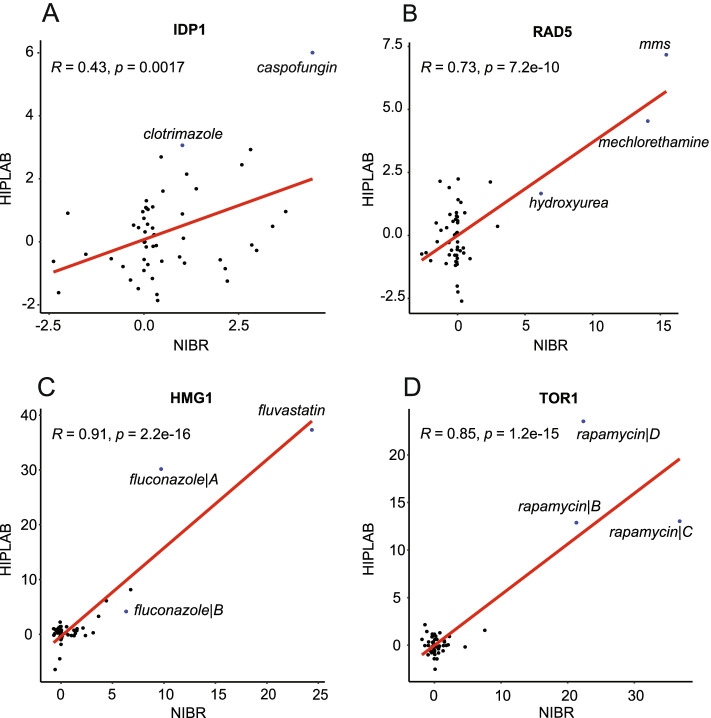
Correlation between gene deletion strains shown for (**A**) *IDP1* (**B**) *RAD5* (**C**) *HMG1* (**D**) *TOR1* between the NIBR and HIPLAB datasets

In addition to measuring the correlation between chemical profiles, the correlation of gene fitness scores across compounds and between datasets can be informative. For this comparison we employed *‘*cofitness’; the degree of similarity between fitness profiles in which the FD scores between two genes are measured across all compounds, using Pearson correlation as a metric. Genes with a high degree of correlation (or cofitness) between profiles are often functionally related. Here, where we have two independent datasets, we expect the same gene to be cofit across the 50 compounds shared between the two datasets. In practice, we observed an overall correlation of ~0.15 for the same gene. Because most genes are not perturbed in any given experiment, we expect that those genes with highly variable scores to exhibit greater correlation. This is indeed the observation; when only significantly sensitive strains are considered (genes with standard deviations in the top 5%), the correlation between genes increases from 0.15 to ~0.5. As the correlation increases, the mechanistic similarity between drugs that significantly perturb a given deletion strain also increases. For example, the *IDP1* gene profiles exhibit a similar pattern of perturbation (R-value ~0.4) (Fig. 2A). Likewise, *RAD5* and *HMG1* exhibit high correlations (R-value ~0.7, ~0.9, respectively) and significant perturbations are seen in mechanistically related compounds such as 1) the DNA-damaging agents’ hydroxyurea, mechlorethamine and methyl methanesulfonate (MMS), and 2) the sterol pathway inhibitors fluconazole and fluvastatin (Fig. 2B, C). Similarly, in the case of *TOR1* (R-value 0.85), fitness deviations arise from a single compound- rapamycin (Fig. 2D).

**Fig. 3:**
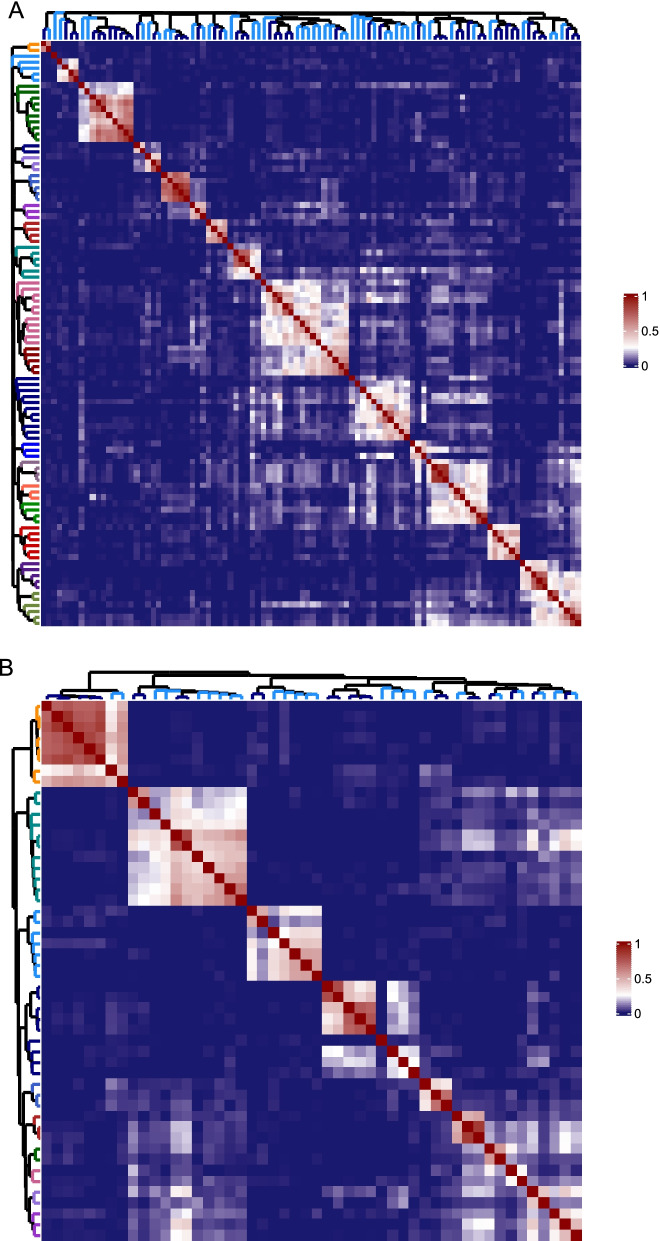
Hierarchical cluster analysis of reference compounds screened by both the HIPLAB and NIBR. To identify robust clusters, we generated the 'coinhibitory' square matrix, defined as the pairwise Pearson correlation between the selected screens, representing the similarity between profiled compounds. Profiles were then hierarchically clustered using (1 - the coinhibitory matrix) as the distance metric and Ward as the agglomeration method. Heatmap of: **A** drugs with established mechanism **B** antimetabolites and DNA-damaging agents. Row dendrogram branches are colored by mechanism of drug action; column dendrogram branches are colored by research institute: NIBR and HIPLAB in navy and light blue, respectively. Drugs within each major cluster represent screens with highly correlated chemogenomic profiles, indicated by both the heatmap color scale and dendrogram height. This suggests that compounds within a cluster act by a similar mechanism. The list of drugs (including the research institutes and the screened doses) in each heatmap from top to bottom and left to right is as follow. Each parenthesis includes drugs corresponding to the colored row dendrogram branches): Panel **A** (NIBR_sphingosine:1.5uM, HIPLAB_sphingosine:6.7uM); (HIPLAB_anisomycin:9.6uM, HIPLAB_cycloheximide:667uM, NIBR_anisomycin:10uM, NIBR_cycloheximide:30nM, NIBR_cycloheximide:50nM); (HIPLAB_curcumin:80uM, HIPLAB_curcumin:90.4uM, NIBR_curcumin:85uM, NIBR_curcumin:55uM, NIBR_curcumin:70uM, NIBR_curcumin:58.5uM, NIBR_curcumin|C:58.5uM, NIBR_curcumin|A:58.5uM, NIBR_curcumin|B:58.5uM, NIBR_curcumin:75uM); (NIBR_myriocin:8.1uM, HIPLAB_myriocin:605nM); (HIPLAB_cerulenin|B:830nM, NIBR_cerulenin:1.6uM, HIPLAB_1,3−diallylurea:8.3mM); (HIPLAB_benomyl:22.9uM, HIPLAB_nocodazole:6uM, NIBR_nocodazole:8uM, NIBR_nocodazole:10uM, NIBR_nocodazole:12uM); (HIPLAB_tofa:1.1uM, NIBR_tofa:500nM, HIPLAB_tofa:880nM); (HIPLAB_tunicamycin:25nM, NIBR_tunicamycin:150nM, NIBR_tunicamycin:200nM, HIPLAB_tunicamycin:200.5nM); (HIPLAB_terbinafine:2.2uM, NIBR_terbinafine:12.4uM, NIBR_naftifine|A:71uM, NIBR_naftifine|B:71uM, HIPLAB_terbinafine:19.9uM, HIPLAB_butenafine:14.7uM); (HIPLAB_clotrimazole:625nM, NIBR_fluconazole:60uM, NIBR_clotrimazole:471nM, NIBR_cyproconazole:280nM, NIBR_fluconazole:30uM, NIBR_voriconazole:530nM, HIPLAB_voriconazole:435nM, HIPLAB_clotrimazole:1.4uM, HIPLAB_fluconazole:33.5uM, HIPLAB_fluconazole:20uM, HIPLAB_myclobutanil:7.7uM); (HIPLAB_itavastatin :7.2uM, HIPLAB_fluvastatin:17.1uM, NIBR_fluvastatin:57.2uM, HIPLAB_atorvastatin:60.2uM, HIPLAB_atorvastatin:78.7uM); (HIPLAB_alverine citrate:130uM, HIPLAB_dyclonine:31.2uM, HIPLAB_alverine citrate:64uM, NIBR_fenpropimorph:273nM, HIPLAB_haloperidol:116.4uM, NIBR_dyclonine:29.2uM, HIPLAB_amorolfine:100uM, HIPLAB_haloperidol:53.2uM, HIPLAB_fenpropimorph:62.5uM, HIPLAB_alverine citrate:93.8uM, HIPLAB_haloperidol:50.8uM); (HIPLAB_tacrolimus:100uM, NIBR_ascomycin:100uM, HIPLAB_tacrolimus:29.8uM); (HIPLAB_nigericin:15.3uM, NIBR_nigericin:8uM, NIBR_nigericin:12uM, NIBR_nigericin:15uM); (HIPLAB_amiodarone:34.4uM, NIBR_amiodarone|B:13.4uM, NIBR_amiodarone|A:13.4uM); (NIBR_chlorpromazine:25.3uM, HIPLAB_trifluoperazine:10.3uM, NIBR_trifluoperazine|A:7.3uM, NIBR_trifluoperazine|C:7.3uM); (HIPLAB_caspofungin:25nM, NIBR_caspofungin:7nM, NIBR_caspofungin|A:10nM, NIBR_ergokonin a:700nM, NIBR_caspofungin|B:10nM, NIBR_ergokonin a:910nM); (HIPLAB_caffeine:993.1uM, NIBR_caffeine:500uM, NIBR_caffeine:1mM, NIBR_caffeine:1.5mM, NIBR_caffeine:2mM); (HIPLAB_rapamycin:1nM, HIPLAB_rapamycin:4nM, HIPLAB_rapamycin:2nM, NIBR_rapamycin:500pM, NIBR_rapamycin:800pM, NIBR_rapamycin:1nM) Panel **B** (NIBR_methotrexate|B:200uM, NIBR_methotrexate|C:200uM, NIBR_methotrexate|D:200uM, NIBR_methotrexate|F:200uM, NIBR_methotrexate|A:200uM, NIBR_methotrexate|E:200uM, UBC_methotrexate:289.6uM, UBC_methotrexate:400uM); (NIBR_ravidomycin:10uM, NIBR_ravidomycin:12.8uM, UBC_epirubicin:18.1uM, UBC_daunorubicin:9.9uM, NIBR_doxorubicin|A:28.1uM, NIBR_doxorubicin|B:28.1uM, UBC_doxorubicin:7.6uM, UBC_doxorubicin:7.7uM, UBC_daunorubicin:22.4uM, UBC_daunorubicin:18.5uM, UBC_doxorubicin:8.1uM); (NIBR_5−fluorouracil:6.9uM, NIBR_flucytosine:8uM, UBC_5−fluorouridine:15.4uM, UBC_5−fluorouracil:457uM, UBC_5−fluorocytosine:1.1uM, UBC_5−fluorocytosine:377nM, UBC_carmofur:3.8uM); (NIBR_6−azauridine|A:200uM, NIBR_6−azauridine|B:200uM, NIBR_mycophenolic:120uM, NIBR_mycophenolic:70uM, NIBR_mycophenolic:100uM, UBC_mycophenolic:780.4nM, UBC_6−azauridine:81.6uM, UBC_mycophenolic:18.9uM, UBC_mycophenolic:32.9uM); (UBC_bleomycin:30.5nM, NIBR_bleomycin:50nM, NIBR_bleomycin:75nM); (UBC_hydroxyurea:18.1mM NIBR_hydroxyurea:8.3mM NIBR_hydroxyurea:16.6mM); (NIBR_mechlorethamine:95uM, UBC_mechlorethamine:29.7uM); (NIBR_mms:10nM, UBC_mms:110mM); (NIBR_aclarubicin:4.9uM, UBC_aclarubicin:5.5uM); (UBC_camptothecin:39.6uM, NIBR_camptothecin:200uM, UBC_camptothecin:424.9nM). Note that live versions of both of these figures are available on our accompanying interactive website Comparative chemogenomics where correlations and relations within and between experiments are more easily visualized

For genes exhibiting cofitness within the 50 compounds shared by both datasets, we observe enrichment for gene pairs in both sets that reflect the mechanistic enrichment of the compounds. For example, 6 of the 50 compounds were DNA-damaging agents, and as a result, several of the top cofit genes were pairs involved in DNA repair. To illustrate this point further, we compared the cofitness of genes perturbed by 22 compounds that had been screened more than once by both groups and which clustered together in the combined dataset. This list included nine classes of drugs: tunicamycin, several azoles, the antimetabolites 5-fluoruracil and methotrexate, nocodazole, 6-azauracil, rapamycin as well as DNA-damaging agents including alkylating agents and the DNA topoisomerase I inhibitor camptothecin. As expected, each dataset clustered by mechanism into the nine classes. Inspecting the top 150 most variable genes in each dataset indicated that ~50% of these genes (73) overlapped. Clustering the genes in the two datasets independently resulted in nine clusters of genes that were enriched for gene function. We found overlapping GO terms and genes for each of these nine clusters demonstrating the shared cofitness between the two datasets (Table 3, Table S1). To examine the agreement between the NIBR and HIPLAB datasets more comprehensively, we combined, and then hierarchically clustered the two HIPHOP datasets together for a subset of mechanistically related compounds (Fig. S2). In the first case we analyzed 98 compounds representing 19 distinct mechanistic classes including TOR signaling, microtubule poisons, *FAS1* inhibitors, cell wall inhibitors, statins, ionophores, ion channel blockers, azoles and morpholine antifungals, (Fig. 3A). In the second case, we analyzed 50 DNA-damaging agents representing ten mechanistic classes including doxorubicin, camptothecin, hydroxyurea, mechlorethamine and MMS (Fig. 3B). In both cases the resulting heatmaps reveal that the screens cluster primarily by the mechanism of drug action and not by the research institute. Notably, screens from NIBR and HIPLAB were interspersed, and all replicates and compounds with the same mechanism clustered together. In the DNA-damaging clustergram, one notable exception was observed for two aclarubicin profiles (one from each research site) that did not cluster with the other anthracycline compounds including doxorubicin, daunorubicin and epirubicin. These differences between specific anthracyclines may reflect true mechanistic differences between these closely related compounds [21, 22]. For example, the individual aclarubicin HIPHOP profiles implicate *RPO31* (encoding an RNA polymerase III subunit) as a potential target whereas the other anthracycline screens do not. In select cases, compounds with similar mechanisms (i.e., part of the same pathway) also clustered together, including the morpholine antifungals, e.g., fenpropimorph and amorolfine, both targeting *ERG2*, the azoles, e.g., fluconazole and clotrimazole, targeting *ERG11*, and the statins, e.g., atorvastatin and fluvastatin, targeting *HMG1*, with all three targets in the sterol biosynthesis pathway. Other examples include clustering of ion channel blockers next to ionophores, amiodarone and nigericin, respectively, and clustering of rapamycin next to caffeine, known to target the TOR pathway in *Saccharomyces cerevisiae* (Fig. 3A). The resulting heatmaps, dendrograms and the correlation between each chemogenomic profile can be viewed live on the accompanying website (Comparative chemogenomics). Table 2 includes the mean coinhibitory values as a function of drug mechanism.

**Table 3:** Cofitness clusters of genes. Common genes between the two studies are italicized.

GO:ID	GO term;	HIPLAB cofit cluster genes	HIPLAB cofit	NIBR cofit cluster genes	NIBR cofit
GO:0030491	DNA heteroduplex formation; camptothecin	CDC31, DML1, FPS1, LSM1, ***MMS4***, ***MUS8***1, ***RAD55***, ***RAD57***, ***RAD59,*** RHO1, RPN4, RSC58, SUP45, YTH1	0.62	CSM3, DDC1, ***MMS4***, ***MUS81***, PPH3, RAD17, RAD54 , ***RAD55***, ***RAD57***, ***RAD59***, RPL26B, RTT101, SAE2, SRS2	0.67
GO:0006289	DNA Nucleotide excision repair; mechlorethamine	***MMS2***, ***RAD1, RAD10***, ***RAD14***, ***RAD2***, ***RAD23***, ***RAD4***, ***RAD5***	0.94	*MMS2*, MPH1, PSO2, ***RAD1***, ***RAD10***, ***RAD14***,RAD18, ***RAD2***, ***RAD23***, ***RAD4***, ***RAD5***, REV1, REV3, WSS1	0.83
GO:0000463	Maturation of LSU-RRNA from tricistronic transcript; 5-fluorouracil	***RRP15***, RRP42	0.99	MAK5, MAS2, ***RRP15***, TRM10	0.95
GO:0046654	Tetrahydrofolate biosynthesis; methotrexate	APM3, CDC21, ***DFR1***, ***FOL2***, *YBT1*	0.76	***DFR1***, FOL1, ***FOL2***, *YBT1*	0.97
GO:0000294	Nuclear transcribed mRNA catabolic process; 6-azauridine	***POP1***, ***POP4***, POP7, RPP1	0.85	AMD1, HAM1, ***POP1***, ***POP4***	0.97
GO:0008202	Steroid metabolism; fluconazole	***ERG11***, FPR1, HMG1, PKC1, RIM101, RIM13, RIM21, RIM9, SNF7, UTR1, YGR122W	0.57	DAP1, ***ERG11***, GCS1, LAA1, MIL1, NCP1, TVP18, UPC2	0.91
GO:0031929	Tor signaling; rapamycin	AVL9, BRL1, CDC20, GLC7, GTR1, GTR2, IKI3, ***KOG1***, KTI12, MEH1, MOD5, NCS2, NCS6, PEP7, RPL34B, RTG2, SER2, SHE4, SLM4, THR4, TIM54, ***TOR1***, ***TOR2***, ***VAM6***, VAM7, VPS27, VPS29, VPS35	0.87	APL5, APL6, APS3, ARF1, CKA2, ERG5, HUR1, ***KOG1***, LSM4, MAK10, MCM2, PHO80, SEM1, ***TOR1***, ***TOR2***, ***VAM6,*** YPP1	0.89
GO:0007021	Tubulin complex assembly; nocodazole	***ARP6***, ***CCT2***, ***CCT4***, ***CCT5***, ***CIN2***, ***PAC2***, ***PAN2***, ***PAN3***, ***SNQ2***, ***SWC3***, ***SWC5***, ***SWR1***, ***TCP1***, ***TUB3***, ***VPS71***	0.85	***ARP6***, BIK1, ***CCT2***, ***CCT4***, ***CCT5***, ***CIN2***, CIN4, ***PAC2***, ***PAN2***, ***PAN3,*** RBL2, ***SNQ2***, ***SWC3***, *SWC5*, ***SWR1***, ***TCP1***, ***TUB3***, ***VPS71***	0.94.
GO:0030968	Endoplasmic reticulum unfolded protein response; tunicamycin	***ALG7***, BCK1, ***CNB1***, ***FYV8***, ***GFA1***, ***HAC1***, ***IRC21***, ***IRE1***, ***NHX1***,***RET2***,***SEC21***,***SEC26***,***SPF1***	0.77	ACK1, ***ALG7***, ***CNB1***, COP1, ***FYV8***, ***GFA1***, ***HAC1***, IPK1, ***IRC21***, ***IRE1***, ***NHX1***, ***RET2***, ***SEC21***, ***SEC26***, ***SPF1***	0.87

### Common response signatures

Our previous global analysis of the HIPLAB dataset [16] revealed that, despite the complexities of pharmacological inhibition, the cellular response to small molecules is limited in the sense that it can be described by a network of 45 major response signatures. These responses comprise chemogenomic profiles with; 1) a characteristic gene signature, 2) distinct GO enrichments, and 3) enriched chemical sub-structures. To test if these response signatures are also present in the NIBR dataset, we used the same methodology as in Lee et al. (2014) [16]; we hierarchically clustered the NIBR screens using coinhibition as a distance metric and a dynamic branch cutting method [23] to generate discrete clusters. 96 robust clusters were initially identified, encompassing ~41% of the profiles. Compared to the 45 major responses in the HIPLAB dataset, the number of NIBR response signatures was two-fold higher. The larger number of responses in the NIBR data may be due to many of these signatures being redundant with respect to their GO enrichments. The NIBR signatures were also longer, as would be expected when clusters are small and when compounds within a cluster are replicates. Indeed, the NIBR screening library contains a large number of replicates (56% of all screens). Accordingly, to minimize redundancy, we modified the dynamic branch cutting parameters to be less sensitive to smaller clusters (see Methods), which resulted in a final set of 42 robust NIBR clusters, a number comparable to the 45 HIPLAB response signatures. In this re-analysis the median number of genes in the response signatures was similar between the final NIBR and HIPLAB signatures (7 vs. 8 genes). Using the overlap coefficient between gene signatures to measure similarity between response types, we found that ~66.7% of the 45 major HIPLAB response types were detectable in the NIBR clusters. These common signatures include iron & copper homeostasis, cell wall signaling, mitochondrial stress, and perturbation of the plasma membrane. More specific responses, often including drugs of known mechanism, included the responses: unfolded protein, anthracycline transcription coupled DNA repair, azoles and statins, ERAD & cell cycle, heme biosynthesis & mitochondrial translocase, *NEO1*-*PIK1*, tubulin folding & SWR complex, superoxide, and DNA damage.

The majority of these conserved chemogenomic response signatures are enriched for biological processes (Table S2). We also provide a tool for the explicit comparison of the HIPLAB and NIBR gene response signatures and their overlap coefficients on the accompanying website (Comparative chemogenomics). Taken together, these results provide further support for the concept that the cellular response to small molecules is finite and that it can be defined by chemogenomic signatures.

Because the comparison of chemogenomic signatures could be influenced by biases in the composition of the screening libraries, we asked which of the final 42 responses were unique to the NIBR dataset. Response signatures that were not detectable in the HIPLAB responses included those comprising the three TOR signaling clusters, the GPCR inhibitor response and the eukaryotic translation initiation factor (eIF) complex inhibitor signature. Additional gene/target-specific responses included: inhibitors of *VRG4*, encoding a Golgi GDP-mannose transporter, *RPL15A* & *SPP41* (ribosome and spliceosome genes) and *FAS1*, encoding fatty acid synthase. We suspect that these responses reflect the NIBR library being enriched for target-focused compounds.

We used the same approach to compare the signatures of the combined dataset to the HIPLAB responses. From a total of 47 responses, ~84% of the original 45 HIPLAB signatures were detected, and of these 38 overlapping signatures, DNA damage responses, as well as the azole & statin, superoxide, tubulin folding & SWR complex, unfolded protein and mitochondrial-specific stress responses were uncovered.  Interestingly, by combining the two datasets, some of the NIBR signatures that had not previously matched a HIPLAB response merged into one of the 38 overlapping responses. Only three signatures were specific to the HIPLAB data: *NEO1*, ubiquinone biosynthesis & proteasome, and the RSC complex & mRNA processing. Conversely, signatures driven by NIBR profiles included: *TIM54*, *RPL15A* & *SPP41*, *VRG4*, eIF, and GPCR inhibitors as well as the major TOR signaling response.

### Target frequency comparison

**Fig. 4:**
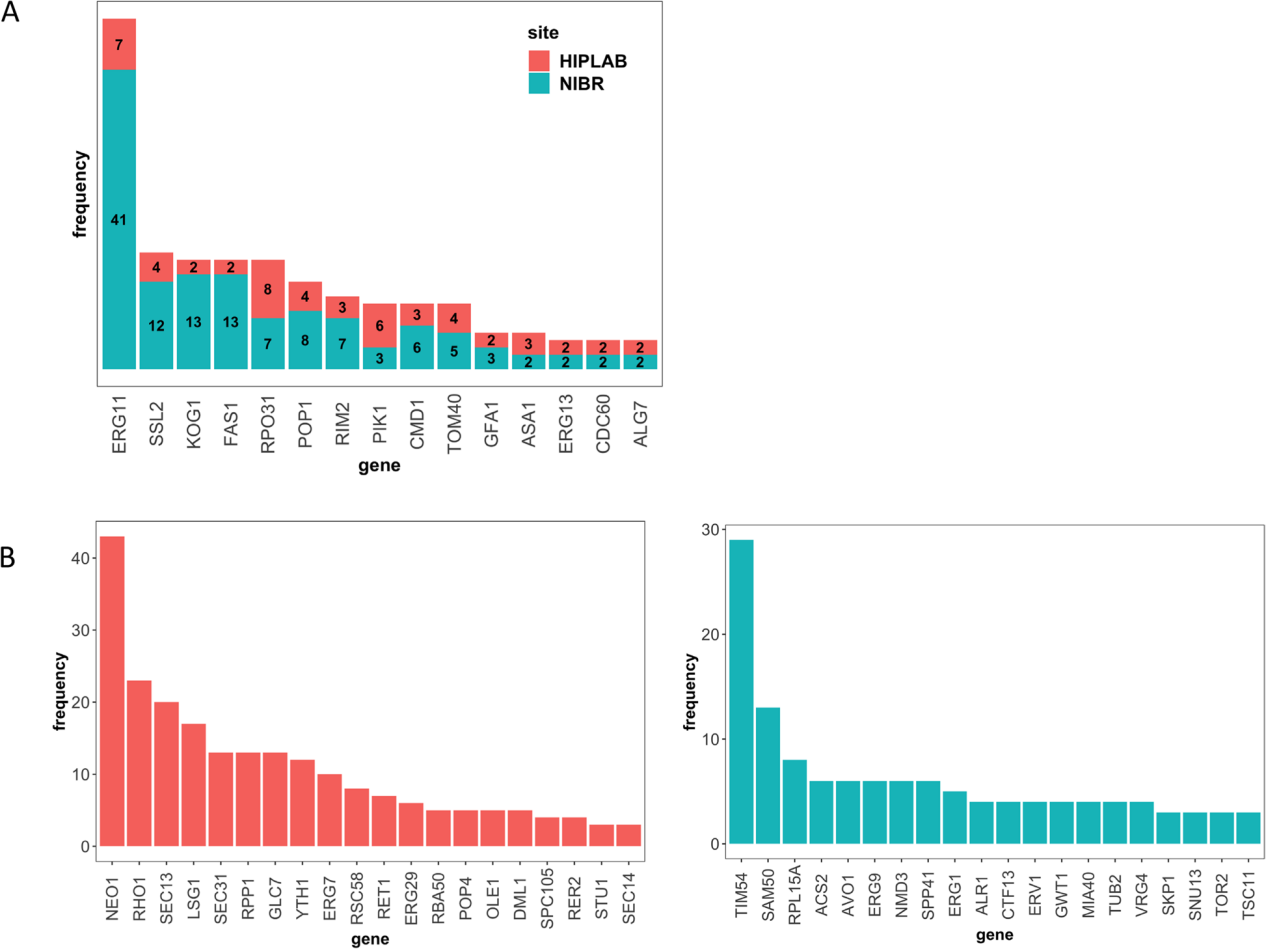
Most frequently targeted genes. **A** Stacked barplot of genes targeted by both HIPLAB and NIBR and their frequency. **B** Genes and their frequency targeted exclusively by left: HIPLAB or right: NIBR

Compared to the HIPLAB dataset, which focused on screening diverse compounds with unknown mechanisms, NIBR clusters were highly enriched for screens identifying specific genes as potential drug targets (Fig. 4). The most frequently identified targets in the NIBR dataset include, 1) *ERG11* (sterol biosynthesis pathway) and *KOG1*, *AVO1*, and *TOR2*, encoding subunits of the Targets of Rapamycin (*TOR1* and *TOR2*) complexes, 2) *FAS1,* encoding fatty-acid desaturase, and 3) the mitochondrial transport gene *TIM54*. The coherence of these signatures suggests that the constituent compounds represent structural analogs or very similar structures. In the azole & statin and *ERG11-GCN* responses, *ERG11* is identified as the target in 31% and 67% of the screens, respectively. In the three rapamycin clusters, the *TOR1 and TOR2* subunits are identified as targets in over half (26) of the 51 screens. Consistent with this hypothesis, in 2012 NIBR published a study of novel Erg11 inhibitors, which suggests that these published inhibitors may be present in the NIBR screening library [24]. Similarly, the high frequency of targeting mTOR (mammalian target of rapamycin) complexes (as evidenced by the three responses associated with TOR signaling) suggests an enrichment of rapamycin analogs in the NIBR compound library. This is consistent with the fact that rapamycin and aging are active areas of inquiry at NIBR [25–27].

HIPHOP profiles presented in studies previously published by NIBR researchers allowed us to infer the structures of selected blinded screens. For example, a Nature Chemical Biology study published by the group demonstrated TIM23-dependent mitochondrial import as the target of the natural product stendomycin [28]. A HIPHOP profile in the NIBR dataset was nearly identical to the published version, (Fig. S3). Similarly, the NIBR published a novel geranylgeranyltransferase inhibitor (uncovering sensitivities of strains encoding subunits of the *CDC43*/*RAM2* heterodimer) that was highly correlated to the HIPHOP profile for NIBR compound 5692 in the NIBR dataset [29] (Fig. S4).

### Compounds and mechanism of action inferred by clustering with reference compounds

**Fig. 5:**
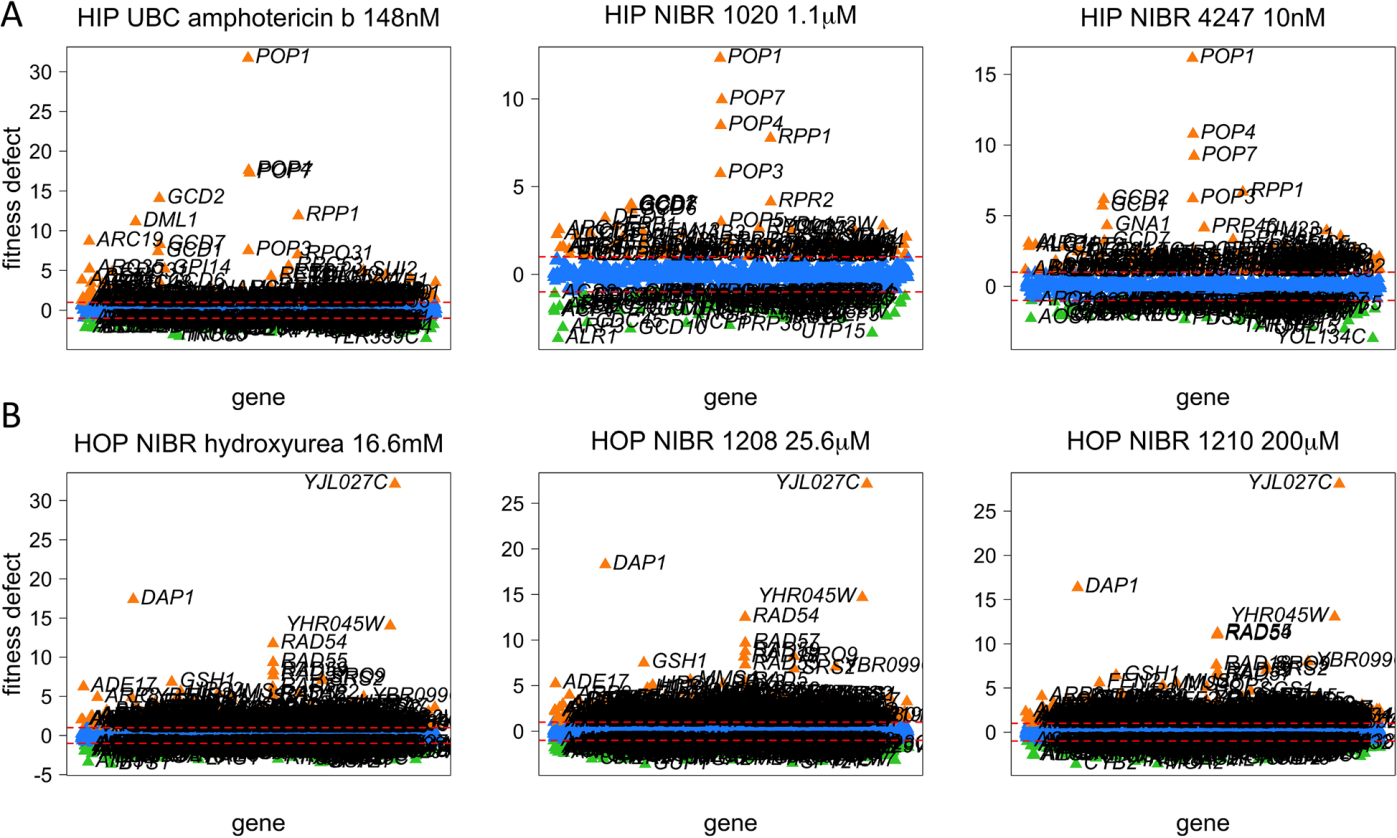
Compounds and mechanisms of action can be inferred by coinhibition with reference compounds. **A** The high coinhibition of the HIPLAB HIP amphotericin chemogenomic profile with two NIBR profiles (4247 and 1020) suggests all three compounds act by the same mechanism. **B** A series of four compounds, 1208, 1209, 1210 and 1211 (only 1208 and 1210 are shown) recapitulated the hydroxyurea chemogenomic profile nearly perfectly suggesting they might be structural analogs

One of the NIBR clusters revealed a potential MoA by virtue of its correlation to reference compounds; the HIPLAB amphotericin B HIP screen was highly correlated with the NIBR 4247 and 1020 HIP screens (> 0.7, *P*-value < 1e-16) (Fig. 5A). In another example, the NIBR compounds 1208, 1209, 1210 and 1211 had correlations of > 0.8 (*P*-value < 1e-16) with HIPLAB hydroxyurea screens, similar to that observed between replicates, suggesting these compounds are in fact both hydroxyurea or closely related structures (Fig. 5B).

## Conclusions

The comparative analysis of the HIPLAB and NIBR datasets provides a systems-level view of the cellular response to small molecules. Considering the enormous complexity of the cell, we find it noteworthy that the ~35 million chemical-genetic quantitative measurements reported here can be described by ~47 chemogenomic signatures, comprising shared chemical structures and biological processes. 60% of these responses were found in an earlier large-scale study [15], and 66.7% in the NIBR dataset. Taken together, 40% of the responses were conserved in all three datasets, suggesting that the proteins encoded by genes within these signatures represent potential starting points for therapeutic intervention. The power of functional genetic screens to uncover drug targets and target pathways, and to delineate the mechanism of action of therapeutics has been demonstrated both in the yeast model system and more recently in meta-analyses of mammalian-cell based CRISPR screens [30–33]. As the complexity of these screens increases (e.g., *in vivo* assays, combined perturbations, etc.), the ability to perform integrated analyses will grow in importance. Based on our analysis of the two largest gene-drug comprehensive datasets collected to date, we show, using standardized protocols and analytics that yeast-based screens can be performed, at scale, across laboratories and that the resulting data are robust.

## Methods

### Source of datasets

The NIBR dataset was downloaded from Hoepfner et al., 2014 [17] through the Drayd digital repository at: 10.5061/dryad.v5m8v. Gene-wise z-scores data of the essential genes present in the heterozygous dataset were selected and combined with the nonessential homozygous dataset for 2725 screens present in both datasets. The HIPLAB data consists of 5905 strains and 3356 screens [16]. For clustering the combined datasets, the two matrices were merged into a final matrix of 5894 strains x 6081 screens. 309 strains in this dataset were absent in the NIBR dataset.

### Identification of significant chemical-genetic interactions

FD scores were calculated for both datasets using slightly different techniques. Specifically, for each HIPLAB strain, log_2_ ratios were calculated for as follows:

1. log_2_ratio_HIPLAB_ = log_2_[<median signal from control samples> / <signal from chemical sample>]

To facilitate comparisons between screens, log_2_ ratios were standardized (separately for heterozygous and homozygous strains). The FD score of strain *i* in screen *j* was computed as follows:

(2) FD_*i,j HIPLAB*_ = (log_2_ratio_*i,j*_ - < median of log_2_ratios for screen *j*>) /<MAD of log_2_ratios for screen *j*>

Because the FD scores follow a standard normal distribution, the probability that a given score is an outlier in this distribution was obtained using a one-tailed *P* test. *P* < 0.001 were identified as significant chemical-genetic interactions. To identify outlier screens for a given deletion strain, FD scores were converted into gene-wise Z-scores and *P*-values [16]. As described [17], the NIBR dataset defined the log_2_ratio roughly as 1/log_2_ratio_HIPLAB_:

1. r_L_ = log_2_ratio_NIBR_ = log_2_[<average signal from chemical sample>/<average signal from control samples>]

the normalized MADL score FD of strain i in screen j was computed as:

(2) MADL_i,j_ = FD_*i,j NIBR*_ = (log_2_ratio_*i,j*_ - < median of log_2_ratios for screen *j*>) /<MAD of log_2_ratios for screen *j*>

The MADL or FD_NIBR_ is roughly equivalent to the negative value of the FD_HIPLAB_.

Lastly, the MADL scores were multiplied with the t-test *P*-value between replicates and the controls to be adjusted for highly variable strains (a_MADL_). The gene-wise z-scores were further estimated using a_MADL_ of strain i over n experiments and the standard deviation (σ) obtained from the middle 70% of the quantiles:

(3) z-score_i_ = a_MADL(i)_/σ_i_

A z-score cutoff of -5 is used to define the significant chemical-genetic interactions [17].

### Identification of HIP hits

‘HIP hits’ are defined as potential targets of profiled compounds with high specificity [16]. In the HIPLAB dataset, ‘clearance’ was defined as a measure of specificity that identifies significant hits in strains exhibiting FD scores greater than zero in a given HIP profile where:

Strains are ordered by FD scores in descending order, where FD_*(i)*_ is the i^th^ greatest FD score in the profile and clearance is defined as the difference between FD scores:

clearance = FD_*(i)*_ – FD_*(i + 1)*_

clearance_*max*_ is the maximum clearance’ associated with the profile, and FD_*max*_ is the FD score of the strain with clearance’ = clearance_*max*_

If any FD_*(i)*_ ≥ FD_*max*_, clearance = clearance_*max*_

otherwise, clearance = clearance’

Clearance thresholds were optimized using the gold standard compounds with known targets and in the dataset resulting in a threshold of 5.75. Therefore, strain(s) with significant FD scores (*P* < 0.001) and clearance_*max*_ ≥ 5.75 are designated HIP hits [16]. We used this clearance scoring system to identify hits in both datasets.

### Hierarchical clustering

Our chemogenomic dataset is in a matrix format where each screen is a column, and each row is a gene (corresponding to its homozygous or heterozygous deletion strain). To identify robust clusters in the NIBR dataset and to fairly compare the two datasets, we followed the same hierarchical clustering methodology used in Lee et al. (2014) [16]. We first replace insignificant scores (standard normal *P* > 0.001) in the NIBR screening matrix with zero, to focus on the most significant cellular responses to chemical perturbation. We then compute coinhibition, the pairwise Pearson correlation between all screens, representing the similarity between the NIBR profiled compounds. Profiles were then hierarchically clustered using (1 – coinhibition) as the distance metric, and the Ward agglomeration method. Discrete clusters were obtained using a dynamic branch [23] cutting method. For the full NIBR dataset, we used the following parameters: deepSplit = 4, minClusterSize = 3, as was done in Lee et al. (2014) [16]. For the final version with 41 clusters, we used deepSplit = 2, cutHeight = 20, minClusterSize = 3. For the combined HIPHOP dataset, we used a minGap = 0.098, deepSplit = 2, minClusterSize = 3.

### Chemogenomic response signatures

In our previous study, we classified HIPHOP cellular response types into chemogenomic signatures defined by characteristic genes and associated biological processes [16]. To determine whether these major response signatures exist in the NIBR dataset, we used the same analytic methods. Specifically, an FD matrix was provided using all profiled compounds as columns and all deletion strains (genes) as rows. Similarity between the cellular responses to the profiled compounds was measured using the Pearson correlation between the matrix columns (coinhibition). The functional similarity between two genes was measured using the Pearson Correlation between the matrix rows (cofitness). To identify robust clusters in the NIBR dataset, profiles were hierarchically clustered using (1 – coinhibition) as the distance metric, and the Ward agglomeration method. For each cluster, we calculated the median FD scores of each deletion strain across all profiles in that specific cluster to generate a median profile. Strains with significantly positive FD scores (standard normal distribution *P* < 0.001) identify the characteristic gene signatures that are an important part of the cellular responses. A standard normal distribution of *P* < 0.001 was used for comparing HIPLAB and NIBR signatures. For the signatures in the combined dataset, we used a threshold standard normal distribution *P* < 0.05.

In the NIBR dataset, 56 clusters were identified and 42 were associated with characteristic genes or gene signatures. Response signatures with fewer than two genes that were not enriched for biological processes were omitted. We performed GO enrichment analysis on each response signature.

Signatures and GO enrichments are available at the accompanying website as well as in Table S2:

Comparative chemogenomics or http://matrika.pharmacy.ubc.ca:3838/ggiaever/Comparative chemogenomics/

## Supplementary Information

**Additional file 1: Figure S1.** Nonessential heterozygous strains are not biologically informative. DNA-damaging agents induced fitness defects in homozygous deletion strains compared to little or no fitness defects in the corresponding nonessential heterozygous deletion strains in response to the same compounds.

**Additional file 2: Figure S2.** Normalized z-scores are not biased by specific signatures. To check for bias in the normalized z-scores for each dataset, we plotted the densities of the top 10% of the signatures vs. all of the signatures (minus this top10%) for the HIPLAB and the NIBR datasets independently. (A) Densities of the z-scores for the HIPLAB dataset (B) Densities of the z-scores for the NIBR dataset.

**Additional file 3: Figure S3.***TIM23* is identified as the target of Stendomycin (aka 5692 in the NIBR dataset) in two different studies despite dosage differences [17, 28]**.**

**Additional file 4: Figure S4.** A geranylgeranyltransferase inhibitor (5692 in the NIBR dataset and compound 1 in Pries et al. (2016)) targets similar genes in two different studies [24, 29].

**Additional file 5: Table S1.** GO enrichment analysis of cofit genes corresponding to Table 3.

**Additional file 6: Table S2.** Overlap coeffiecients for HIPLAB ➔ NIBR gene response signatures and NIBR ➔ HIPLAB gene response signatures and GO enrichments for all HIPLAB and NIBR gene response signatures.

## Data Availability

The HIPLAB dataset analyzed in the current study is available for query and in a variety of downloadable formats at http://chemogenomics.pharmacy.ubc.ca/HIPHOP/. The raw microarray data are archived in the ArrayExpress database (www.ebi.ac.uk/arrayexpress) under accession no. E-MTAB-2391 [6]. The Novartis Institute for Biomedical Research dataset analyzed during the current study is available in the Drayd digital repository at 10.5061/dryad.v5m8v [7].

## Abbreviations

NIBR: Novartis Institute of Biomedical Research
MoA: Mechanism of Action
HIPHOP: HaploInsufficiency Profiling and HOmozygous Profiling
FD: Fitness Defect
MMS: Methyl methanesulfonate
eIF: Eukaryotic initiation factor
GO: Gene Ontology
O.D.: Optical Density
