# Supplementary figures and images for: The cellular response to drug perturbation is limited: comparison of large-scale chemogenomic fitness signatures
Source: BMC Genomics. 2022 Mar 11;23:197. doi: 10.1186/s12864-022-08395-x (PMC8915488; doi:10.1186/s12864-022-08395-x)

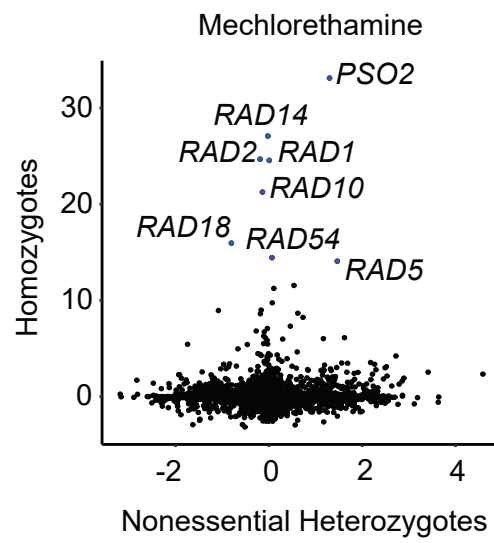

Supplement: Supplementary file 1 — Additional file 1: Figure S1. Nonessential heterozygous strains are not biologically informative. DNA-damaging agents induced fitness defects in homozygous deletion strains compared to little or no fitness defects in the corresponding nonessential heterozygous deletion strains in response to the same compounds. [file 12864_2022_8395_MOESM1_ESM.pdf]

**density of z-scores HIPLAB**

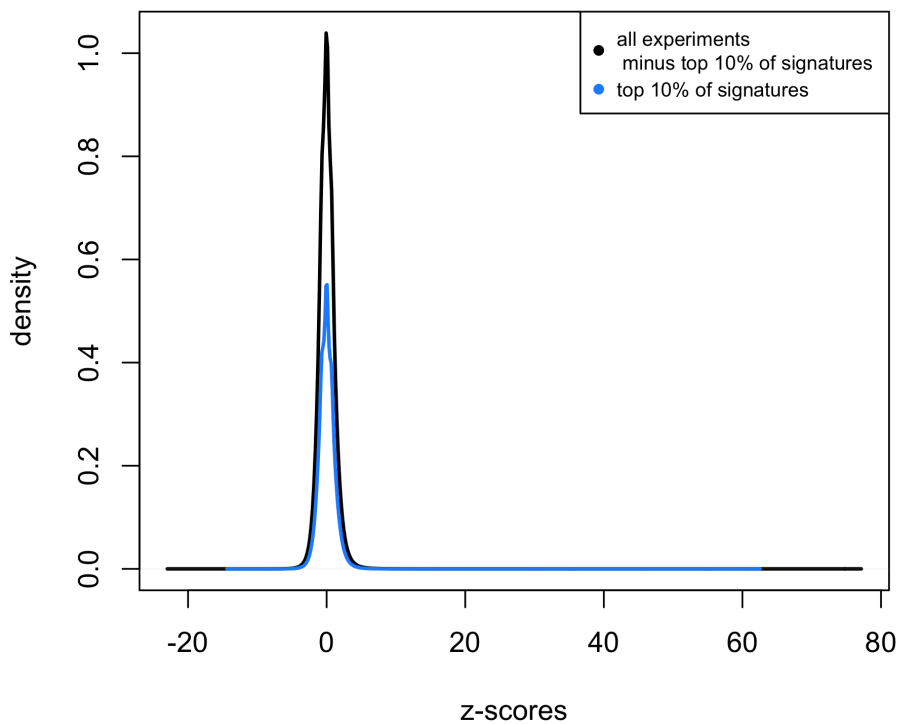

**density of z-scores NIBR**

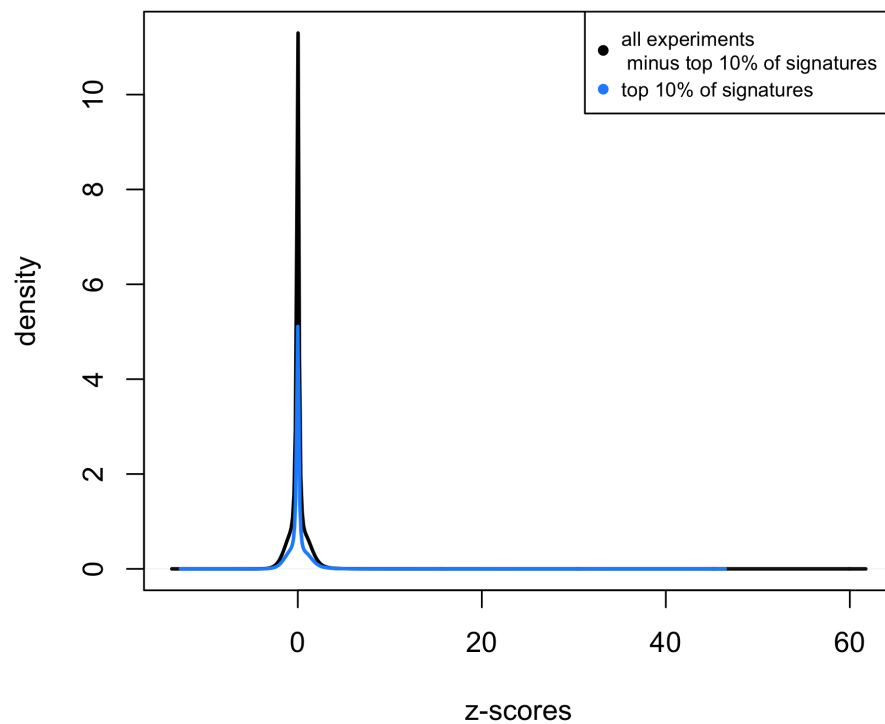

Supplement: Supplementary file 2 — Additional file 2: Figure S2. Normalized z-scores are not biased by specific signatures. To check for bias in the normalized z-scores for each dataset, we plotted the densities of the top 10% of the signatures vs. all of the signatures (minus this top10%) for the HIPLAB and the NIBR datasets independently. (A) Densities of the z-scores for the HIPLAB dataset (B) Densities of the z-scores for the NIBR dataset. [file 12864_2022_8395_MOESM2_ESM.pdf]

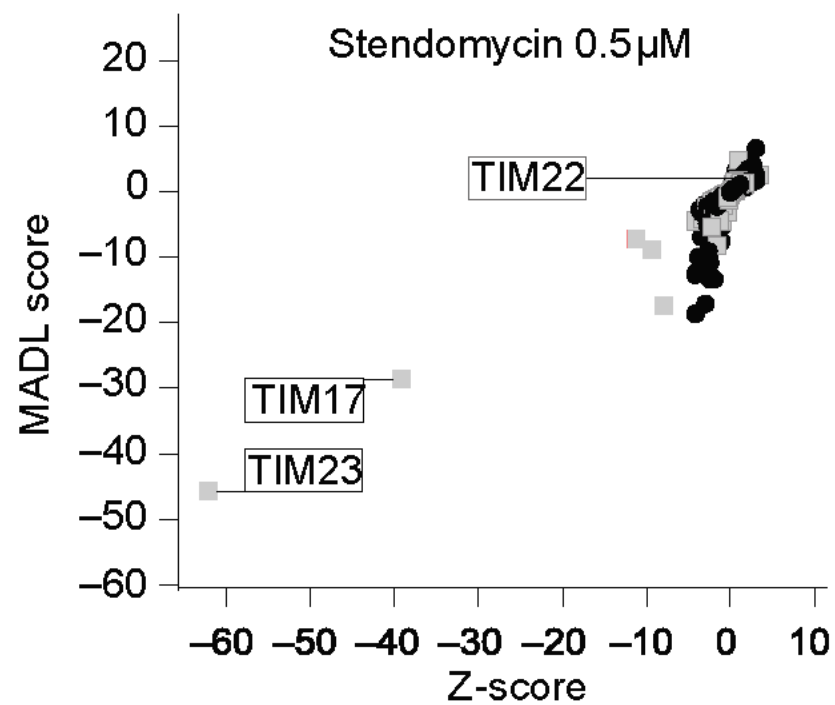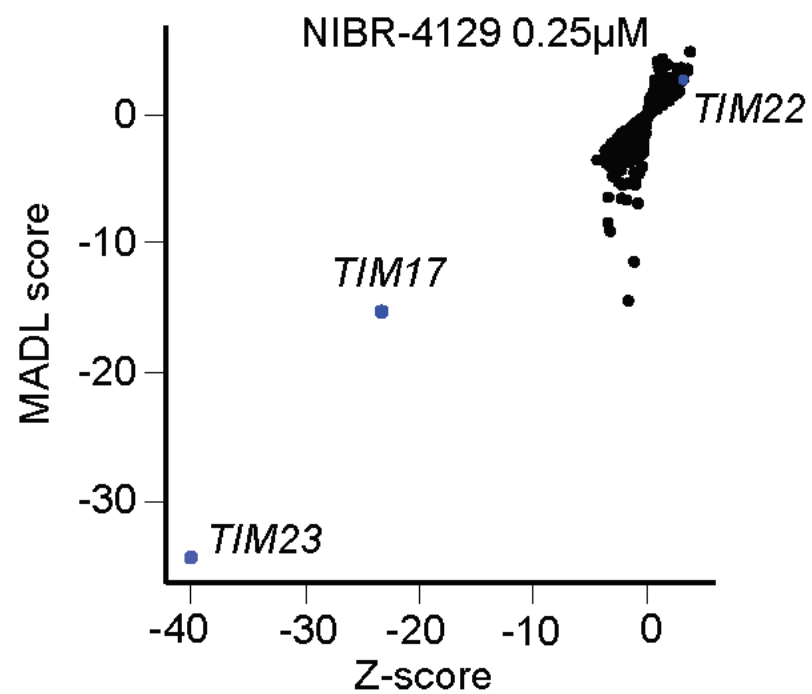

Supplement: Supplementary file 3 — Additional file 3: Figure S3.TIM23 is identified as the target of Stendomycin (aka 5692 in the NIBR dataset) in two different studies despite dosage differences [17, 28]. [file 12864_2022_8395_MOESM3_ESM.pdf]

HIP profile Compound 1

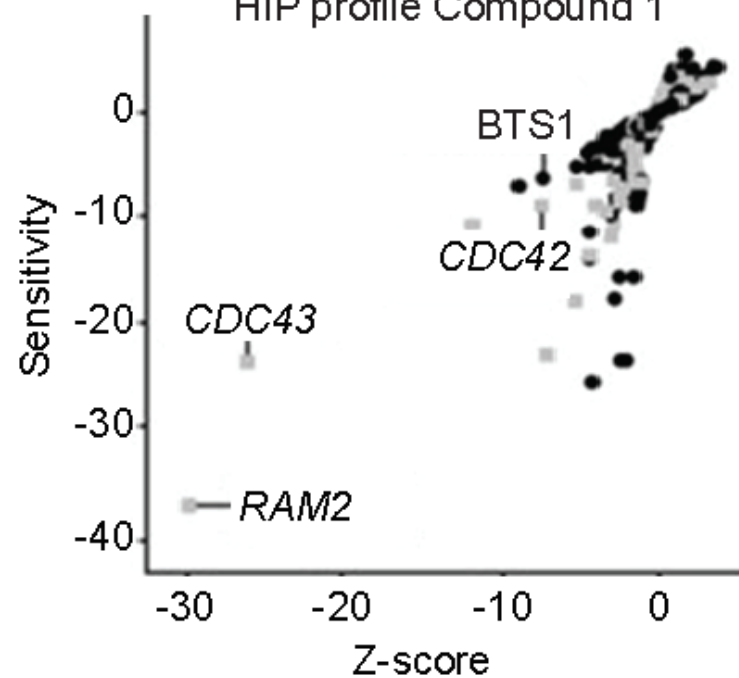

NIBR-5692 10 $\mu$ M

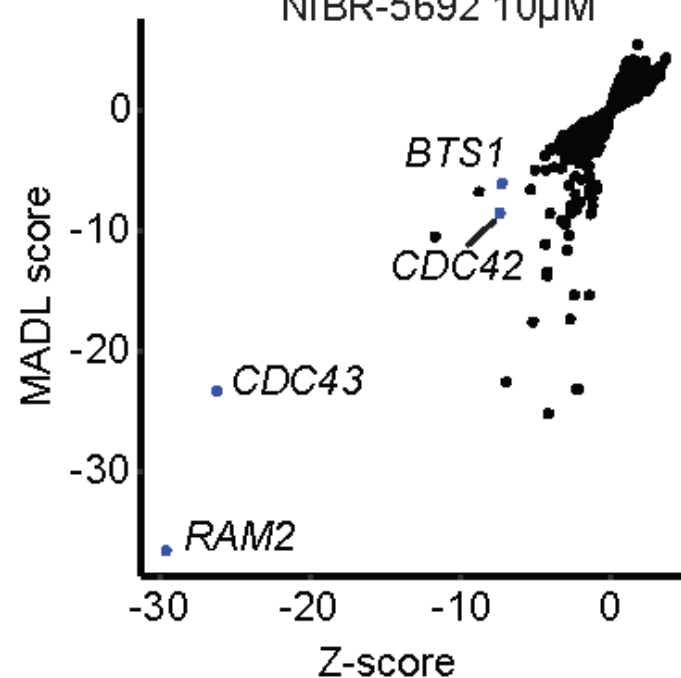

Supplement: Supplementary file 4 — Additional file 4: Figure S4. A geranylgeranyltransferase inhibitor (5692 in the NIBR dataset and compound 1 in Pries et al. (2016)) targets similar genes in two different studies [24, 29]. [file 12864_2022_8395_MOESM4_ESM.pdf]
